# Clinical knowledge, health policies and social identities. Commentary on Lara Rzesnitzek (2013) “Early psychosis as a mirror of biologist controversies in post war German, Anglo-Saxon and Soviet psychiatry”

**DOI:** 10.3389/fpsyg.2014.00202

**Published:** 2014-03-18

**Authors:** Nicolas Henckes

**Affiliations:** Centre de Recherche Médecine, Sciences, Santé, Santé mentale et Société (CERMES3), CNRSVillejuif, France

**Keywords:** early diagnosis, schizophrenia, mental health policy, psychiatric profession, experience of illness

In her wide ranging and thoughtful article Rzesnitzek (2013), recalls the long, conflicting and at times convoluted history of attempts to describe and delineate the beginnings of schizophrenia. As Rzesnitzek shows us, this history is at once scientific, clinical, and political. It involved practitioners from all over the Western world as well as from what used to be called the Eastern world. The reader is struck by the range of the debates, the number of conflicting positions as well as the size of the research effort involved in these debates. In a way, the debates over the beginnings of schizophrenia were merely an abridged version of those over the nature of the disorder itself. In fact, while Rzesnitzek herself does not clearly make this point, her article demonstrates quite convincingly that the debates over the beginnings of schizophrenia *were* debates over the very definition of the disorder. What was seen as the manifestation of an early form of schizophrenia or what was rather understood as a predisposing condition clearly reflected divergent visions of the nature and symptoms of the disorder. As a sociologist and social historian, I would add that these visions should in turn be associated with the settings and practical conditions in which psychiatrists saw patients.

What also necessarily strikes the reader is the open-ended nature of these debates. There clearly were—and probably still are—too many uncertainties to overcome to reach an agreement over criteria for defining the early phases of schizophrenia. Given these uncertainties, one may and probably should wonder what stimulated the interest of several generations of psychiatrists in developing tools and criteria for the early diagnosis of schizophrenia. Rzesnitzek does not give an answer to this question, although her article suggests the fascination that may have been created among researchers by questions surrounding the nature of what a recent book has called “the sublime object of psychiatry” (Woods, 2011). In the remainder of this commentary, I prefer to reflect on some of the consequences of the debates for the people concerned. I will specifically comment on two dimensions of the story of early psychosis, which Rzesnitzek does not explore at length.

The first concerns the policy and political dimensions of the story. Seen from Germany, this may not be an important perspective. Nazism, with its programs of sterilization and euthanasia, brought into disrepute both a generation of psychiatrists and ways of thinking about mental disorders, which probably prevented any further efforts at developing preventive practices and policies in the field of mental health in Germany after World War II. However, in many countries and in various ways, both mental hygiene and eugenicist movements remained strong players in the psychiatric field until well after 1945 (Kevles, 1985; Grob, 1991; Rose, 2001; Bashford and Levine, 2010). The idea of mental health as a resource to be preserved and of mental health professionals as contributors to the public good have been major aspects of psychiatric thinking in most Western countries after World War II. In many countries, these ideas have translated into programs in primary prevention, especially with children (Jones, 1999; Stewart, 2013). On a darker side, sterilization programs continued in the US as well as in Northern Europe until at least the late 1970s (Broberg and Roll-Hansen, 2005; Largent, 2008). To what extent researchers in the field of early diagnosis or genetic psychiatry aimed at contributing to these policies is not clear, just as it is not clear what sort of prevention practices have been developed from their research.

Today's practitioners in the field of early intervention have developed a strong awareness of the policy implications of their work, and a segment of the field has even developed a commitment to developing policies that may help to better screen, diagnose, and treat schizophrenia in its early phase. The International Declaration on Youth Mental Health launched in 2012 by a group of youth psychiatrist is the last and most spectacular action in this direction (Coughlan et al., 2013). Yet these actions rely on a concept of mental health policy which differs in major ways from earlier proposals. While the mental hygiene movement was built upon the idea that the psychiatrist was the only expert in defining and enforcing prevention practices, today's mental health movement insists on the necessary participation of the people concerned in their own treatment and care. In a way, concern for mental health affects all of us, and may be a way of life for the most vulnerable. The psychiatric profession is only one actor in the drama of mental health, and often doesn't play the most important role.

This leads to my second commentary, which concerns transformations in the experience of developing schizophrenia as it relates to the changing experiences of being young and becoming adult. The history of schizophrenia as a “coming-of-age” disorder remains largely to be written. Yet, as Rzesnitzek reminds us, age is an obvious component of this disorder. The label for the most iconic form of schizophrenia, “hebephrenia,” was historically proposed by German psychiatrists Kahlbaum and Hecker to label a disorder typically characterized as affecting young people (Kraam and Phillips, 2012). The term was not kept in DSM III, but the definition of schizophrenia in this manual entailed as a criterion an age of onset before 45—a criterion, however, that was removed in subsequent editions (American Psychiatric Association, 1980). More recently, the concept of an “at-risk mental state” targets young people between 16 and 30 (McGorry et al., 2003). And in fact, current practices and policies of early intervention are built upon the premise that young adulthood is an age of maximal vulnerability to mental health disorders.

There is nothing here to surprise a sociologist. As a large body of scholarship in sociology and psychology has now shown, the age between 20 and 30 has emerged as a new life phase characterized as an age of uncertainties, both existential and economic (Booth et al., 1999; Arnett, 2001; Van de Velde, 2008; Booth, 2012). At the same time, it is clear that for most young people these uncertainties, however distressing they might be, will never translate into a problem as dramatic as a major psychiatric disorder. The characterization of early psychosis as a condition affecting young people should be understood in this context. In fact, many mental health professionals see a continuum between minor mental health problems that may develop as a consequence of the existential turmoil of young adulthood, and major psychiatric disorders—or, at least, they are not able to differentiate between a minor mental health problem and the initial signs of what may turn out to be a major psychiatric disorder (see for instance Patrick McGorry's model of clinical staging: McGorry et al., 2007). In turn, it is probable that early intervention practices and policies will affect our vision of young adulthood—including young people's visions of themselves. In North America, several social movements initiated and led by young people are now trying to make a case for youth mental health on academic campuses, and a new generation of youth mental health activists has emerged who are beginning to play an important role in advocating early intervention. In this regard, resilience to early psychosis is perhaps becoming a way for a new generation of young people to build their identity.

Both these discussions point to the fact that medical entities have a social life that extends well beyond the jurisdiction of medicine. As medicine legitimates the existence of a phenomenon as a medical entity, it also leaves open the possibility for many other actors to use this definition for their own purpose—whether these actors are politicians, administrators, activists or patients. This is why discussions on the dangers of psychiatric labeling and on ethical safeguards to help psychiatrists anticipate and prevent the consequences of their judgment may not necessarily always be effective. What patients and society at large do with medical concepts usually goes beyond what medical men and women—including ethicists—imagine. However conscious of the implications of their judgments medical practitioners may be, what becomes of these judgments will certainly be far beyond their reach. Philosopher Ian Hacking has proposed the concept of “looping effect” to describe the transformations which people labeled with a medical diagnosis may in turn create within these diagnostic labels once they have adopted them as their own (Hacking, 1995, 1999). Historian Charles Rosenberg also wrote about the “tyranny of diagnosis” to point to both the necessity of diagnosis in medical practice and the burden of its often unwanted and unexpected consequences (Rosenberg, 2002). This may be even more complicated for the medical profession in situations such as early intervention where the medical status of a category remains disputed, although this category nevertheless has a life of its own outside medicine.

A final striking dimension of the story told by Rzesnitzek is the existence of local variations in the conceptualization and use of a diagnostic category such as early psychosis. This aspect would probably have been even stronger if Rzesnitzek had had the chance to describe actual research and clinical practices that have developed around this category in different countries at different periods. Indeed, such variations not only reflect different clinical traditions, but also different approaches to the practical issue of treating people and different approaches to psychiatric research. The very German history told by Rzesnitzek has a lot to do with the specificity of psychiatric research in that country and its organization around the psychiatric clinic as an academic institution. In contrast, the funding of US psychiatric research by the American Congress may have made it much more sensitive to pressures from the social world. However, from my two previous series of remarks, one could also infer that these variations also owe much to the diverse ways in which the people concerned act in relation to their labels. This layer of complexity adds to those already present in Rzesnitzek's article.
